# One‐Year Quality of Life Among Survivors of Hospitalization for Omicron Infection in Brazil: A Multicentre Prospective Cohort Study

**DOI:** 10.1002/jmv.70687

**Published:** 2025-11-18

**Authors:** Geraldine Trott, Marciane Maria Rover, Fernando Luis Scolari, Mariana Motta Dias da Silva, Denise de Souza, Rosa da Rosa Minho dos Santos, Raíne Fogliati De Carli, Emelyn de Souza Roldão, Gabriela Soares Rech, Duane Mocellin, Jennifer Menna Barreto de Souza, Aline Paula Miozzo, Carolina Rothmann Itaqui, Gabrielle Nunes da Silva, Sergio Renato da Rosa Decker, Erica Neves Leite, Carlos Delmar do Amaral Ferreira, Lucas Gobetti da Luz, Gabriel Beilfuss Rieth, Lucas Tramujas, Fernando Azevedo Medrado, Bruna Fornazieri Piotto, Gizelle Fernanda Oliveira Silva, Josafá Ferreira Chaves, Saionara Cristina Francisco, Precil Diego Miranda de Menezes Neves, Victor Augusto Hamamoto Sato, Viviane Cordeiro Veiga, Kaique Lima Martins, Cláudio Dornas de Oliveira, Sabrina Gomes dos Santos, Juliana Cardozo Fernandes, Thiago Costa Lisboa, Vivian Menezes Irineu, Mauricio Antonio Pompilio, Adriana de Oliveira França, Aline Coletto Jaccottet, Juliana Carvalho Schleder, Vinicius Ortigosa Nogueira, Vandack Nobre, Daniel Souto Silveira, Cézar Eumann Mesas, Diego Miltersteiner, Emanuelle Toledo Ortiz, Fernando Gioppo Blauth, Luciane Maria Facchi, Milena Soriano Marcolino, Ana Carolina Peçanha Antonio, Paulo R. Schvartzman, Bruna Brandão Barreto, Caroline Cabral Robinson, Maicon Falavigna, Carisi Anne Polanczyk, Luiz Antonio Nasi, Regis Goulart Rosa, Post‐Covid Brasil 1 Group Autorship

**Affiliations:** ^1^ Projects Office Hospital Moinhos de Vento Rio Grande do Sul Brazil; ^2^ Cardiology Department Hospital Moinhos de Vento Porto Alegre Rio Grande do Sul Brazil; ^3^ Postgraduate Program in Cardiology Medical School/Universidade Federal do Rio Grande do Sul Porto Alegre Rio Grande do Sul Brazil; ^4^ Research Institute Hospital Moinhos de Vento Porto Alegre Rio Grande do Sul Brazil; ^5^ Research Unit, Inova Medical Porto Alegre Rio Grande do Sul Brazil; ^6^ Hospital Moinhos de Vento Porto Alegre Rio Grande do Sul Brazil; ^7^ Nephrology Department Hospital Moinhos de Vento Porto Alegre Rio Grande do Sul Brazil; ^8^ HCor Research Institute São Paulo São Paulo Brazil; ^9^ Hospital Metropolitano Dr. Célio de Castro Belo Horizonte Minas Gerais Brazil; ^10^ Internal Medicine Department Hospital Alemão Oswaldo Cruz São Paulo São Paulo Brazil; ^11^ Hospital Beneficência Portuguesa de São Paulo São Paulo Brazil; ^12^ Hospital Santa Casa BH Belo Horizonte Minas Gerais Brazil; ^13^ Hospital Ernesto Dornelles Porto Alegre Rio Grande do Sul Brazil; ^14^ Hospital de Clínicas de Porto Alegre Porto Alegre Rio Grande do Sul Brazil; ^15^ Hospital Doutor Leo Orsi Bernardes Itapetininga São Paulo Brazil; ^16^ Hospital do Coração de Mato Grosso do Sul Campo Grande Brazil; ^17^ RS Heart Institute Fundação Universitária de Cardiologia Porto Alegre Rio Grande do Sul Brazil; ^18^ Center for Studies, Research, and Human Development Hospitais da Universidade Estadual de Ponta Grossa Ponta Grossa Paraná Brazil; ^19^ Tropical Medicine Research Center (CEPEM) Porto Velho Rondônia Brazil; ^20^ Hospital de Clínicas Universidade Federal de Minas Gerais Belo Horizonte Minas Gerais Brazil; ^21^ Hospitalize Clinical and Population Research Center Guaíba Rio Grande do Sul Brazil; ^22^ Hospital Universitário de Londrina Londrina Paraná Brazil; ^23^ Hospital Universitário de Canoas Canoas Rio Grande do Sul Brazil; ^24^ Hospital Beneficência Portuguesa de Ribeirão Preto São Paulo Brazil; ^25^ Associação Hospitalar Vila Nova Porto Alegre Rio Grande do Sul Brazil; ^26^ Department of Clinical Medicine, Medical School Universidade Federal de Minas Gerais Belo Horizonte Minas Gerais Brazil; ^27^ Institute for Health Technology Assessment Universidade Federal do Rio Grande do Sul Porto Alegre Rio Grande do Sul Brazil; ^28^ Intensive Care Unit Hospital da Mulher – Maria Luzia Costa dos Santos Salvador Bahia Brazil; ^29^ Internal Medicine Department Hospital Moinhos de Vento Porto Alegre Rio Grande do Sul Brazil; ^30^ Medical School Universidade Federal do Rio Grande do Sul Porto Alegre Rio Grande do Sul Brazil

**Keywords:** Brazil, COVID‐19, Omicron variant, post‐acute COVID‐19 syndrome, quality of life, SARS‐CoV‐2

## Abstract

The lingering effects of COVID‐19 may impair survivors' long‐term health‐related quality of life (HRQoL). However, multicentre studies focusing on the Omicron variant, particularly in middle‐ and low‐income settings, are scarce. We conducted a prospective cohort study across 24 Brazilian hospitals between December 2021 and March 2024, when Omicron was the predominant variant. Adult COVID‐19 survivors were followed for 12 months via telephone interviews. The primary outcome was HRQoL at 12 months, assessed using the EuroQol five‐dimension three‐level questionnaire (EQ‐5D‐3L; range −0.17 to 1.0). All‐cause mortality was a secondary outcome. Among 649 participants (47.9% women; median age 71 years), the median EQ‐5D‐3L utility score at 12 months was 0.69 (IQR 0.41–0.80), and survival was 86.6%. Factors associated with lower HRQoL included female sex, age ≥ 60 years, Charlson comorbidity index ≥ 1, and need for respiratory support. Mortality was associated with age ≥ 60 years (HR 2.81), CCI ≥ 2 (HR 2.17), need for low‐flow oxygen therapy (HR 2.03), and BMI ≥ 25 kg/m² (HR 0.58). One year after hospitalization, Omicron survivors in Brazil showed impaired HRQoL and high mortality. Older age, comorbidities, and need for oxygen therapy were linked to worse outcomes. Long‐term support strategies are needed for these vulnerable populations.

## Trial Registration

The study was registered in the ClinicalTrials. gov database (ID: NCT05165979, registered on December 12, 2021).

**Abbreviations**
AI: artificial intelligence
BMI: body mass index
CCI: Charlson Comorbidity Index
CI: confidence interval
CVD: cardiovascular disease
ECMO: extracorporeal membrane oxygenation
HR: hazard ratio
HRQoL: health‐related quality of life
ICU: intensive care unit
IQR: interquartile range
MV: mechanical ventilation
NIV: noninvasive ventilation

## 1 Introduction

COVID‐19 has surpassed 700 million cases worldwide, with more than 30 million positive tests and 700,000 deaths in Brazil alone [1]. While new confirmed cases and deaths have decreased substantially, reflecting vaccination rates exceeding 60% globally and 85% nationally [1, 2, 3], the physical, mental, and cognitive marks left by COVID‐19 make previous infection a latent issue in the health scenario [4, 5].

Despite the growing understanding of COVID‐19, major gaps remain in the literature regarding its long‐term impact on health‐related quality of life (HRQoL) and survival among patients who required hospitalization. While most studies have focused on short‐term outcomes or high‐income populations, evidence from middle‐ and low‐income settings is limited, especially in the context of high vaccination coverage and the Omicron variant [6, 7, 8], which has distinct clinical characteristics compared to earlier strains. Additionally, few prospective studies have comprehensively evaluated predictors of HRQoL and survival over extended periods after hospitalization for COVID‐19 [9]. Addressing these knowledge gaps is crucial to inform patient management and resource allocation strategies in diverse healthcare systems.

This study aimed to evaluate factors associated with HRQoL at 12 months among patients who survived hospitalization for COVID‐19 in Brazil when Omicron was the predominant variant. Secondary objectives included analyzing 1‐year survival and HRQoL at three, six, and 9 months after hospital discharge.

## 2 Methods

### 2.1 Study Design and Follow‐Up

The rationale and design of the Post‐COVID Brazil 1 Study (NCT05165979) have been published previously [10]. Briefly, this is a multicentre prospective cohort study of adults surviving hospitalization for COVID‐19, recruited from 24 hospitals in Brazil and included between December 2021 and March 2023. Survivors were followed for up to 1 year (until March 2024) through structured and centralized telephone interviews conducted at 3, 6, 9, and 12 months after hospital discharge. For participants with communication difficulties, interviews were conducted with their proxy (a responsible family member who was instructed to answer from the patient's perspective). The institutional review boards of all participating centers approved the study. Written informed consent was obtained from participants or their proxies at the time of enrollment during hospital stay.

### 2.2 Participants

This study included patients aged 18 years or older, who presented clinical symptoms consistent with COVID‐19, required hospitalization for a confirmed SARS‐CoV‐2 infection for a period greater than or equal to 48 h, and survived to hospital discharge. Confirmed COVID‐19 diagnosis required a positive reverse transcription polymerase chain reaction or antigen detection test for SARS‐CoV‐2, collected up to 14 days before the index hospitalization. Exclusion criteria included life expectancy less than 3 months due to any underlying condition, absence of a family member accompanying a patient with communication disabilities (e.g., aphasia, cognitive deficits, or non‐Portuguese speakers), absence of telephone contact, withdrawn consent, or previous inclusion in this study.

### 2.3 Associated Factors

Three sets of independent variables were evaluated: (1) sociodemographic data (age, sex, self‐identified race, years of schooling, and family income); (2) pre‐COVID‐19 health status (smoking status, alcohol abuse, medical comorbidities, body mass index [BMI], history of mental health illness [previous diagnosis of anxiety or depression], and COVID‐19 vaccination history); and (3) COVID‐19 episode severity (severity during hospital stay, thromboembolic events, length of hospital stay, intensive care unit [ICU] admission, and length of ICU stay). Age was categorized as under 60 years and 60 years or older. The Charlson Comorbidity index (CCI) was used to assess the burden of comorbidities, stratified as 0, 1, and 2 or more. BMI was categorized as less than 25 and 25 or more. Cardiovascular disease (CVD) was defined as heart failure, coronary artery disease with or without myocardial revascularisation, moderate‐to‐severe valve disease, resistant hypertension (three or more antihypertensive drugs), permanent arrhythmias (atrial fibrillation/flutter), or cardiomyopathies. The severity of the acute infection was defined as the worst condition during hospital stay according to the highest score on a modified six‐category ordinal scale [11], as follows: 1–not admitted to hospital; 2–admitted to hospital but not requiring oxygen therapy; 3–admitted to hospital but requiring low‐flowoxygen therapy; 4–admitted to hospital requiring high‐flow nasal cannula or noninvasive ventilation (NIV); 5–admitted to hospital requiring mechanical ventilation (MV) or extracorporeal membrane oxygenation (ECMO); and 6–death. Patients with a severity score of 1 or 6 were not included in this study.

### 2.4 Outcomes

#### 2.4.1 Primary Outcome

The primary outcome was the HRQoL utility score, assessed at 12 months after discharge from the index hospitalization for COVID‐19 using the EuroQol five‐dimension three‐level questionnaire (EQ‐5D‐3L). The EQ‐5D‐3L consists of a descriptive system comprising five dimensions that assess the patient's mobility, self‐care, usual activities, pain/discomfort, and anxiety/depression at three levels of severity (no problems, some problems, and extreme problems). Scores are derived using country‐specific value sets that reflect societal preferences for health states. In the Brazilian population, EQ‐5D‐3L scores range from −0.17 (where zero is a health state equivalent to death; negative values are worse than death) to 1 (indicating the best health state, with no problems at all) [12]. The estimated minimal clinically important difference for the EQ‐5D‐3L is 0.03 [13], and the mean value in the Brazilian population is 0.82 [14]. Patients who died during follow‐up were assigned a score of zero on all subsequent follow‐ups.

#### 2.4.2 Secondary Outcomes

The secondary outcomes included HRQoL at 3, 6, and 9 months, the incidence of death and associated factors at 12 months after discharge from the index hospitalization for COVID‐19, the exploration of the cause of death, and the persistence of symptoms during long‐term follow‐up. To ensure an accurate and reliable classification of outcomes, all deaths were adjudicated by two independent medical experts who reviewed audio recordings of telephone interviews with family members. The cause of death and its relationship to COVID‐19 were adjudicated through consensus. Adjudicators also evaluated whether each death was associated with COVID‐19 based on clinical criteria, including the time interval between infection and death, the primary cause of death (e.g., oncologic, pulmonary, cardiovascular, or other causes), and the presence of significant reported sequelae. Any discrepancies in adjudication were resolved through discussion between the two adjudicators. Data on pre‐hospitalization quality of life were collected during the first follow‐up assessment to establish the patient's temporal context.

### 2.5 Statistical Analysis

Continuous variables were assessed for the normality of data distribution using the Shapiro‐Wilk test and visual inspection of histograms. Categorical variables are presented as counts and percentages, while continuous variables are presented as mean (SD) for normally distributed data and median (IQR) for non‐normally distributed data.

For the 12‐month HRQoL assessment, missing data were imputed for participants who had at least one previous follow‐up assessment completed (31 cases). Missing values were imputed for each item of the EQ‐5D‐3L questionnaire using the mice package. For participants without any EQ‐5D‐3L information 1 month before COVID‐19, data were imputed using ordinal logistic regression based on sex, age, race, CCI, and severity score (41 cases). The mode was imputed for those with missing data for only some observations (6 cases).

The HRQoL (EQ‐5D‐3L) score was calculated using the eq. 5d package. The association between quality of life and independent variables was analyzed using generalized estimating equations (GEE) and reported as Beta coefficients with 95% confidence intervals (CI). The Kruskal‐Wallis test was used to evaluate differences in quality of life across the five evaluation timepoints. To assess consistency, a sensitivity analysis was performed for factors associated with quality of life at 12 months, excluding participants who died. The survival function was calculated, and overall survival curves were constructed using the Kaplan‐Meier estimator of overall survival. Univariate Cox models were used to assess the significance of covariates, and Kaplan‐Meier curves were constructed for significant variables. Log‐rank test results were included to assess significant differences between the curves. A multivariate Cox model was computed including age, CCI, BMI, and severity score. The Schoenfeld test was used to assess the proportional hazards assumption, and multicollinearity was assessed by calculating the variance inflation factor (VIF). All statistical analyzes were performed using R version 4.3.1 (R Foundation for Statistical Computing) [15].

Artificial intelligence (AI)‐assisted writing tools were used to support the writing process of this manuscript for language refinement and drafting suggestions. The final article was critically reviewed and edited by the authors to ensure accuracy and compliance with academic standards.

## 3 Results

**Figure 1:**
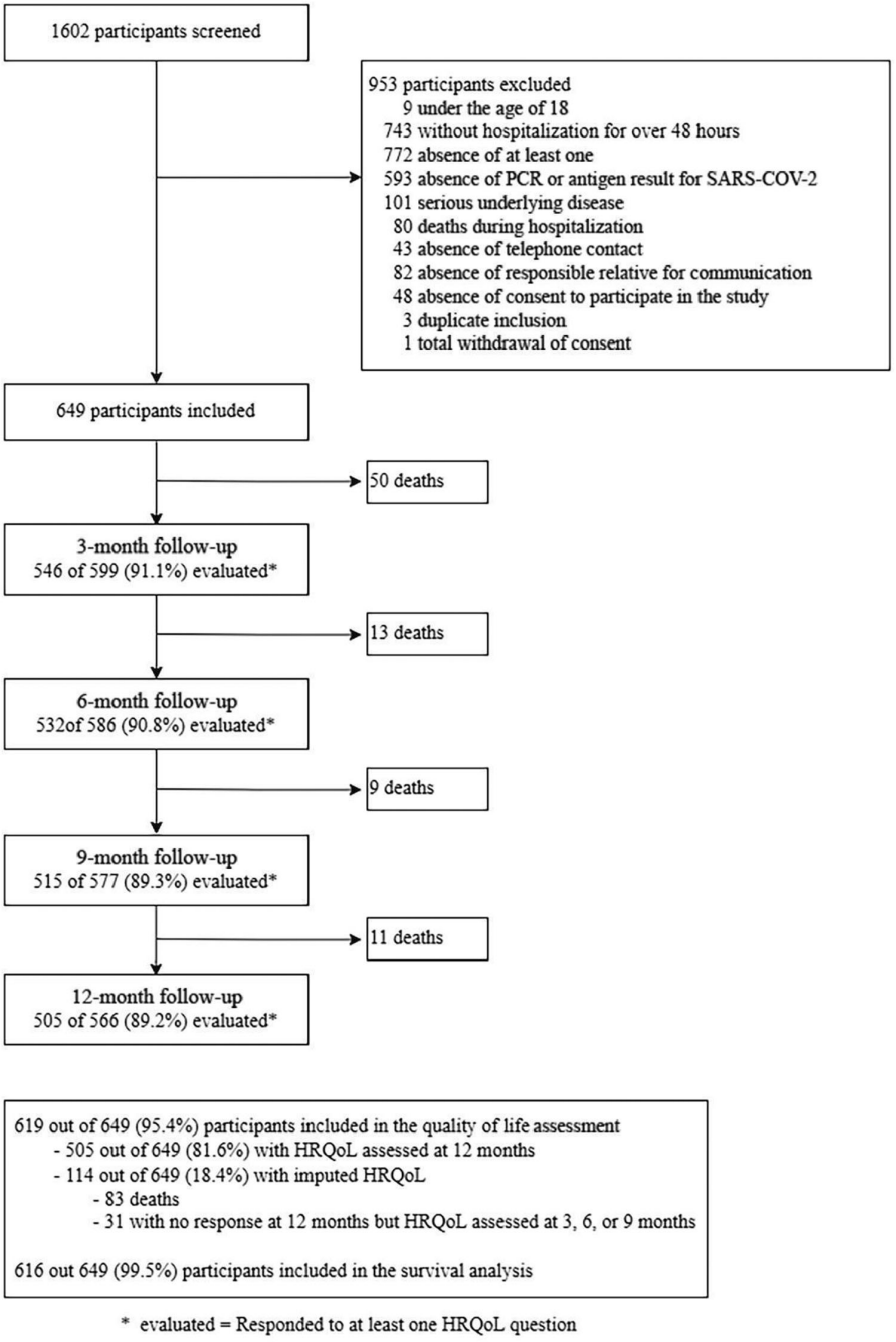
Flow diagram of post‐hospitalization survivors of COVID‐19.

A total of 649 participants were recruited from 24 hospitals in Brazil (Supporting e‐Figure 1). Of these, 83 (12.8%) died before completing 1 year of follow‐up, 53 participants (8.2%) withdrew consent during long‐term follow‐up, and 505 (77.0%) were assessed at the 12‐month follow‐up, which was completed in March 2024. Thus, primary outcome data from 619 participants were included in the final analysis (Figure 1).

### 3.1 Participant Characteristics

Table 1 shows the baseline characteristics of the participants. The median age was 71 years (IQR 60‐80), and 322 participants (52.0%) were men. The most common comorbidities were CVD in 356 (57.5%), systemic arterial hypertension in 280 (45.2%), history of anxiety or depression in 215 (34.7%), and diabetes in 210 (33.9%), with 257 participants (41.5%) having a CCI ≥ 2. The most prevalent symptoms at study entry were cough in 495 (80.0%), fever in 345 (55.7%), and dyspnea in 288 (46.5%). Based on the highest COVID‐19 severity scale score during hospital stay, participants were categorized as follows: 288 (52.8%) score 2 (admitted to hospital but not requiring oxygen therapy); 146 (26.8%) score 3 (admitted to hospital but requiring low‐flow oxygen therapy–nasal cannula or simple face mask); 86 (15.8%) score 4 (admitted to hospital requiring ventilatory support by NIV or high‐flow nasal cannula); and 25 (4.6%) score 5 (admitted to hospital requiring MV or ECMO). Given the small number of participants requiring MV, categories 4 and 5 were pooled into a single group for analysis. No patient required ECMO. The median length of hospital stay was 8 days (IQR 5‐13). Differences between the groups of patients included and not included in the analysis are detailed in Supporting Information e‐Table S1.

**Table 1:** Characteristics of participants recruited for the study.

	Total (*N* = 619)
Sex	
Female	297/619 (47.98)
Age	
Under 60 years	154/619 (24.88)
Greater than or equal to 60 years	465/619 (75.12)
Greater than or equal to 85 years	92/619 (14.86)
Median (IQR)	71.00 (60.00–80.00)
Self‐identified race^1^	
White	489/619 (79.00)
Non‐White	130/619 (21.00)
Years of schooling, median (IQR)^2^	12.00 (7.00–16.00)
Smoking^3^	
Current smoking	43/619 (6.95)
Past smoking	267/619 (43.13)
No history of smoking	309/619 (49.92)
Alcohol abuse^4^	90/619 (14.54)
Average family income	
Up to R$2000	96/517 (18.57)
R$2001 to R$4000	112/517 (21.66)
R$4001 to R$10,000	121/517 (23.40)
R$10,001 to R$20,000	96/517 (18.57)
Above R$20,000	92/517 (17.79)
BMI	
BMI ≥ 25	362/619 (58.48)
Median (IQR)	25.97 (23.05–30.23)
CCI	
0	179/619 (28.92)
1	183/619 (29.56)
≥ 2	257/619 (41.52)
Median (IQR)	1.00 (0.00–2.00)
Comorbidities^5^	
Cardiovascular disease	356/619 (57.51)
Systemic arterial hypertension	280/619 (45.23)
History of mental illness	215/619 (34.73)
Diabetes	210/619 (33.93)
COPD, asthma, or pulmonary fibrosis	137/619 (22.13)
Solid tumor with and without metastasis	80/619 (12.92)
Use of immunosuppressants	53/616 (8.60)
Moderate‐to‐severe chronic renal insufficiency	53/619 (8.56)
Transplant (solid organs/bone marrow)	30/619 (4.85)
Leukemia	18/619 (2.91)
AIDS	3/619 (0.48)
COVID‐19 vaccination history^6^	573/618 (92.72)
Complete	522/575 (90.78)
Incomplete	53/575 (9.22)
Worst severity scale score during hospital stay	
Without oxygen therapy or ventilatory support (score 2)	288/545 (52.84)
Low‐flow oxygen therapy (score 3)	146/545 (26.79)
Nasal cannula oxygen or NIV or MV (scores 4 and 5)	111/545 (20.37)
Need for MV	25/618 (4.05)
Need for NIV	61/618 (9.87)
Need for high‐flow nasal cannula	64/618 (10.36)
Need for low‐flow oxygen	233/539 (43.23)
Thromboembolic event during hospitalization	25/618 (4.05)
Length of hospital stay, median (IQR)	8.00 (5.00–13.00)
ICU admission	115/618 (18.61)
Length of ICU stay, median (IQR)	5.00 (2.00–10.00)

*Note:* Data are n/n total (%) unless otherwise specified.

Abbreviations: BMI, body mass index; CCI, Charlson comorbidity index; COPD, chronic obstructive pulmonary disease; ICU: intensive care unit; IQR, interquartile range (p25–p75); MV, mechanical ventilation; NIV, noninvasive ventilation.

^1^ Non‐white: those who self‐identified as Black, Brown, Yellow, or Indigenous.

^2^ Years of schooling are assessed without considering failing grades.

^3^ Current smoking: having smoked at least 1 cigarette in the past 30 days.

^4^ Alcohol abuse: 14 drinks per week for women and 21 drinks per week for men.

^5^ Cardiovascular disease: heart failure, coronary artery disease with or without myocardial revascularisation, moderate/severe valve disease, resistant hypertension (3 or more antihypertensive drugs), permanent arrhythmias (atrial fibrillation/flutter), or cardiomyopathies; History of mental illness: preadmission diagnosis of anxiety or depression.

^6^ Incomplete = No vaccine dose or only one dose of Pfizer, CoronaVac, or AstraZeneca. Complete = At least one dose of Janssen or at least two doses of the other vaccines.

### 3.2 One‐Year Quality of Life

**Figure 2:**
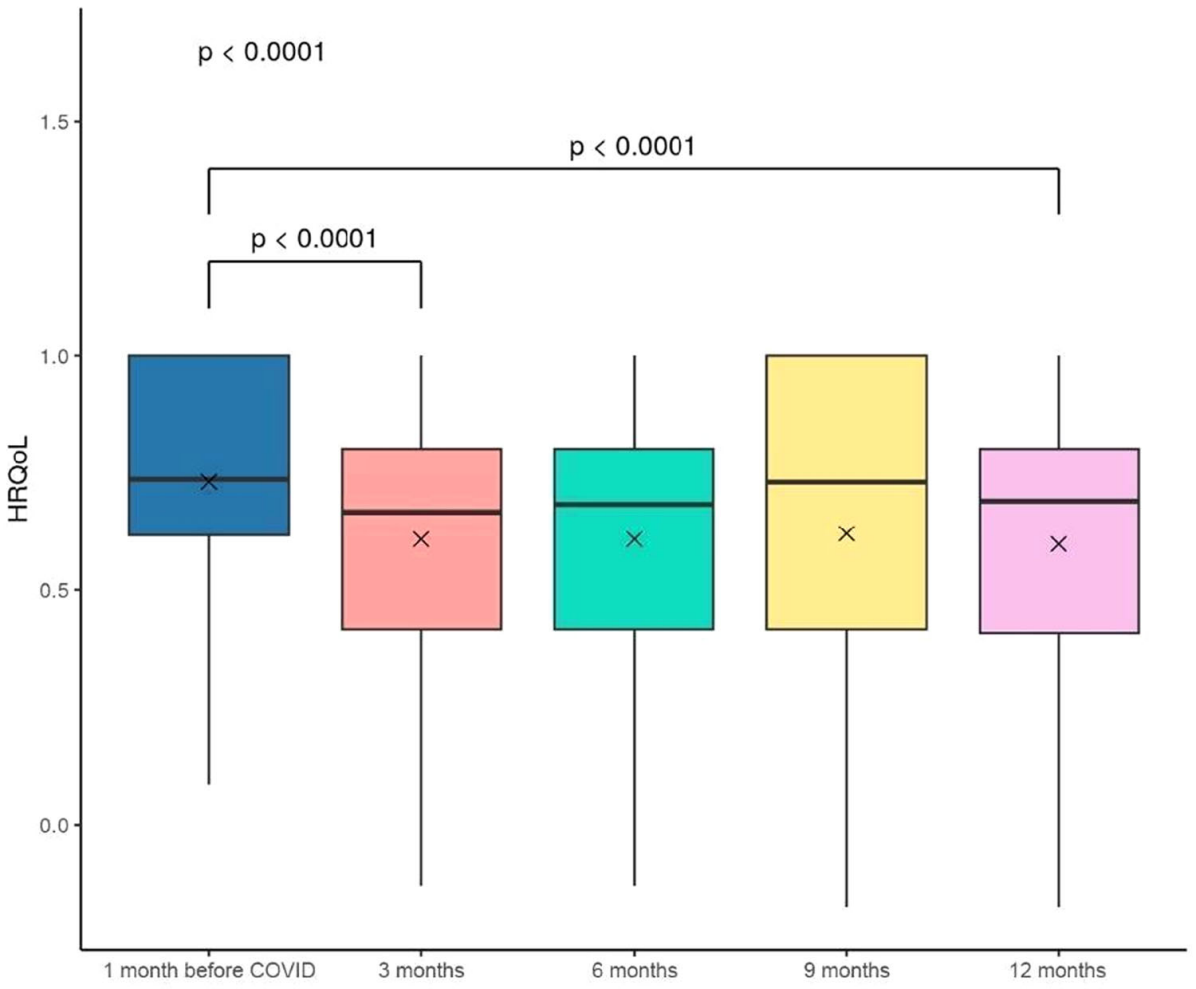
Participants EQ‐5D‐3L utility score during the follow‐up.

All 619 participants with available data for the EQ‐5D‐3L were included in the primary outcome analysis. The HRQoL score 1 month before the index hospitalization for COVID‐19 was 0.74 (IQR 0.62–1.00). During follow‐up, the median score decreased to 0.67 (IQR 0.42–0.80) at 3 months, 0.68 (IQR 0.42–0.80) at 6 months, 0.73 (IQR 0.42–1.00) at 9 months, and 0.69 (IQR 0.41–0.8) at 12 months (Figure 2). At the end of 12 months, participants experienced worsened levels of mobility, self‐care capacity, capacity for usual activities, intensity of pain and discomfort, and levels of anxiety and depression, compared to the baseline levels. Specifically, at 12 months, 6.9% of individuals were unable to walk, 8.0% had limitations in self‐care, 10.8% were unable to perform their usual activities, 40.1% had some degree of pain or discomfort, and 47.7% reported moderate‐to‐extreme anxiety or depression (Supporting Information e‐Figure S2; e‐Table S2).

### 3.3 Factors Associated With One‐Year Quality of Life

**Figure 3:**
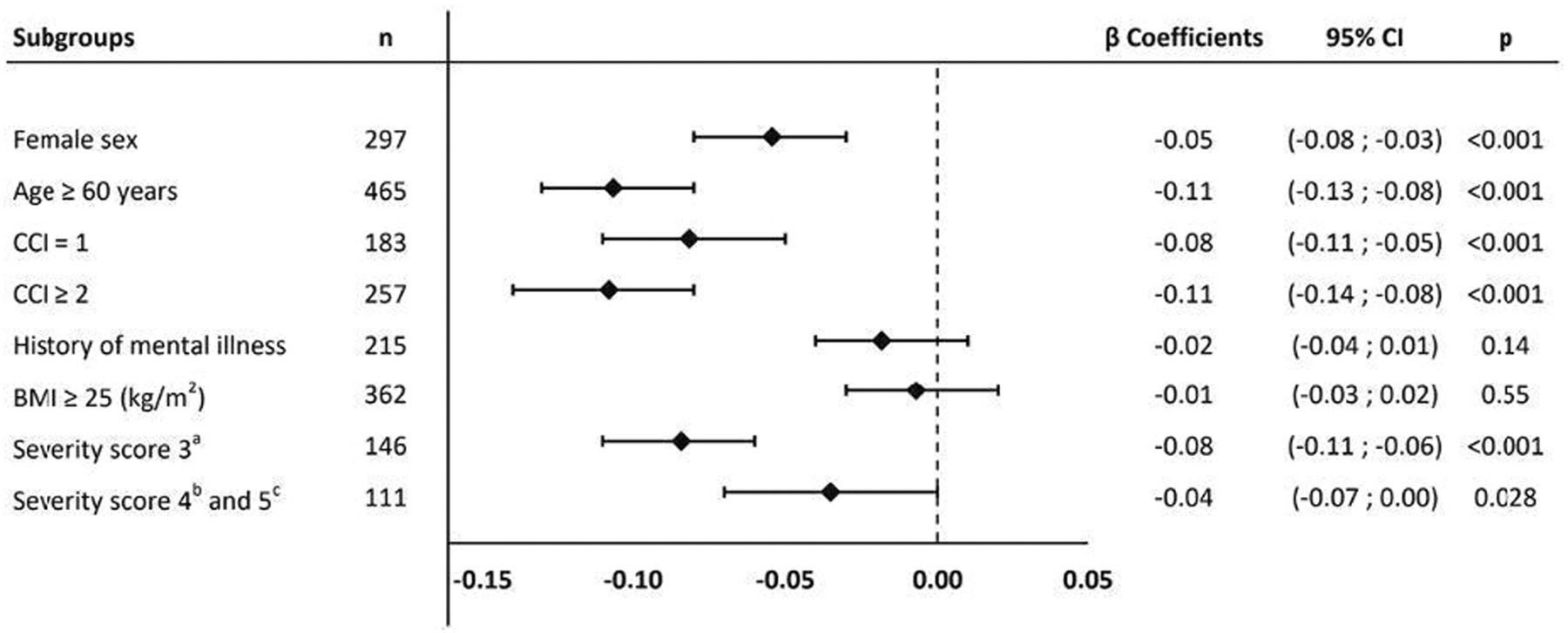
Multivariate generalized estimating equation (GEE) analysis of factors associated with quality of life at 12 months.

The following factors were identified as associated with a decline in HRQoL at 12 months: female sex (β, −0.05 [95% CI −0.08; −0.03]), age ≥ 60 years (β, −0.11 [95% CI −0.13; −0.08]), CCI = 1 (β, −0.08 [95% CI −0.11; −0.05]), CCI ≥ 2 (β, −0.11 [95% CI −0.14; −0.08]), need for low‐flow oxygen therapy (severity score 3) (β, −0.08 [95% CI −0.11; −0.06]), and need for high‐flow nasal cannula oxygen, NIV, or MV (severity scores 4 and 5) (β, −0.04 [95% CI −0.07; 0], *p* = 0.03). History of mental illness and BMI ≥ 25 were not associated with poorer HRQoL at 12 months (Figure 3). Supporting Information e‐Figure S3 presents a comparison of quality of life at 12 months between participants who required ICU admission and those who did not. The results of sensitivity analysis were consistent with those of the main analysis (Supporting Information e‐Figure S4).

### 3.4 Survival Analysis

Survival rates were 91.9%, 89.8%, 88.4%, and 86.6% at 3, 6, 9, and 12 months, respectively. The most common causes of death were pulmonary (32.5%), cancer‐related (20.5%), and cardiovascular (16.9%) complications, with 41.0% of deaths being classified as directly or indirectly related to COVID‐19 (Supporting Information e‐Figure S5; e‐Figure S6; e‐Table S3).

**Figure 4:**
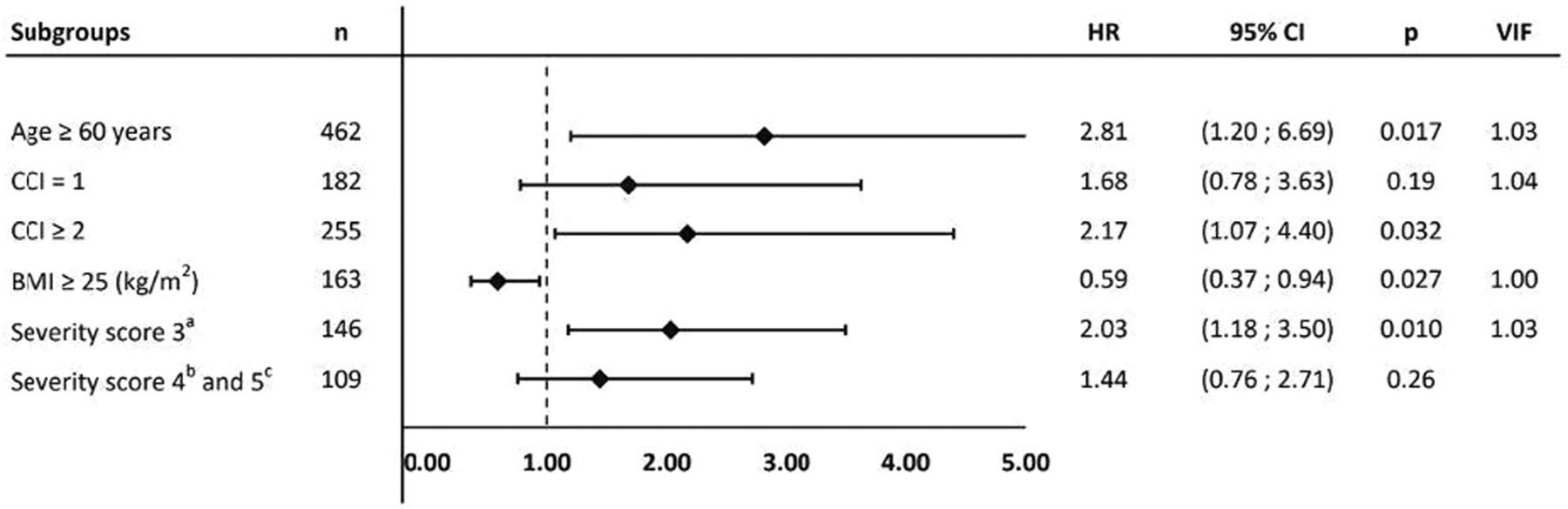
Multivariate Cox regression analysis of factors associated with mortality at 12 months.

**Figure 5:**
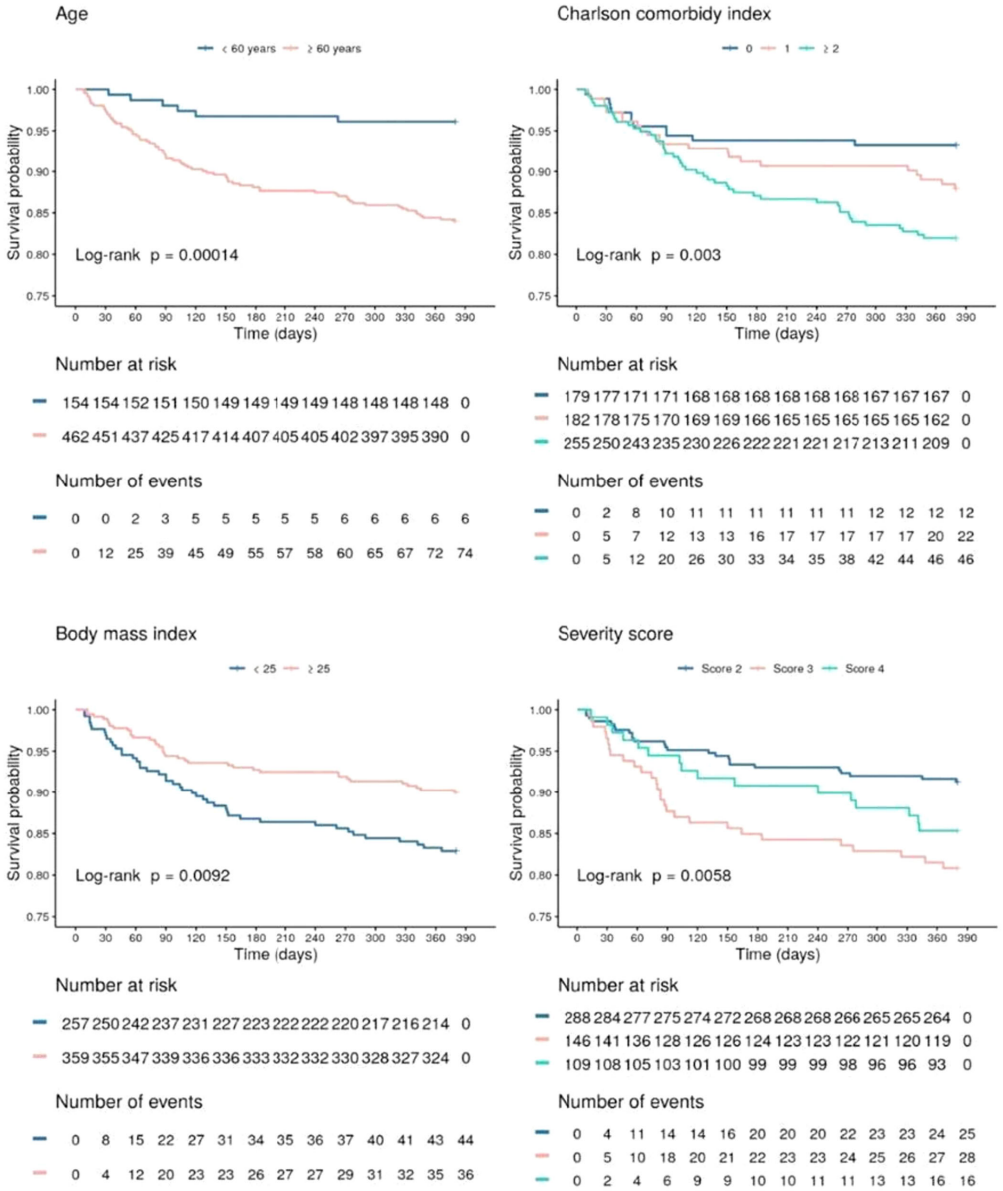
Kaplan‐Meier survival curves for comparison groups.

In adjusted analysis assessing mortality, age ≥ 60 years (hazard ratio [HR] 2.81 [95% CI 1.20; 6.59], *p* = 0.02), CCI ≥ 2 (HR 2.17 [95% CI 1.07; 4.40], *p* = 0.03), and low‐flow oxygen therapy (HR 2.03 [95% CI 1.18; 3.50], *p* = 0.01) were associated with worse survival (Figure 4). BMI ≥ 25 appeared as a protective factor for survival (HR 0.59 [95% CI 0.37; 0.94], *p* = 0.03). The survival curves comparing the groups are presented in Figure 5, and the univariate regression analyzes are provided in Supporting Information e‐Table S4.

## 4 Discussion

In this multicentre prospective cohort study conducted among adult survivors of hospitalization for COVID‐19 during the Omicron era in Brazil, HRQoL at 1 year was lower than the baseline level before the index hospitalization. Additionally, the 1‐year survival rate was 86.0%. Older age, comorbidities, and need for respiratory support during the index hospitalization were associated with worse 1‐year quality of life and survival.

Previous studies have demonstrated a decline in quality of life following COVID‐19 hospitalization. However, these studies vary considerably in terms of sample size, follow‐up duration, and context, as many were conducted before widespread vaccination and the emergence of the Omicron variant [16, 17, 18, 19]. Moreover, research from middle‐ and low‐income countries is limited. Our findings indicate that patients surviving hospitalization for COVID‐19 experienced an important reduction in HRQoL scores 12 months after hospitalization, compared to the period before hospitalization. This reduction was evident in the domains of mobility, self‐care, usual activities, intensity of pain and discomfort, and levels of anxiety and depression. This decline was also observed at 3, 6, and 9 months, with the most pronounced reduction at the 3‐month follow‐up. These results align with previous research. One study found that up to 9 months after hospital discharge, patients exhibited lower HRQoL compared to the general population, particularly in physical dimensions [7]. Another study reported an even more pronounced decline in patients who had been admitted to the ICU and required MV [16, 17].

Our study cohort consisted mostly of older adults with significant comorbidities, reflecting a profile of heightened vulnerability to SARS‐CoV‐2 infection. Poorer HRQoL after 12 months was associated with factors such as age over 60 years, female sex, CCI ≥ 1, and need for oxygen therapy or ventilatory support (including low‐flow oxygen, NIV, or MV). Survival analysis further identified older age, need for low‐flow oxygen therapy, and CCI ≥ 2 as independent risk factors for shorter survival. Increasing age is associated with physical and functional decline, which can compromise quality of life among survivors of various diseases, including COVID‐19 [19, 20, 21]. Furthermore, older age is a recognized predictor of severity, supporting its importance as a critical risk factor for unfavorable clinical outcomes [22, 23]. Regarding sex, women affected by COVID‐19 had lower scores in domains such as physical functioning and body pain up to 1 year after hospital discharge [7, 24]. This is consistent with studies reporting lower quality of life among women, potentially due to factors such as chronic pain, heightened inflammation, mood disorders, and the disproportionate mental health burden experienced during the COVID‐19 pandemic [24].

The ongoing decline in quality of life after COVID‐19 may also be associated with the exacerbation of comorbidities. Comorbidity burden, measured by the CCI, has been an independent predictor of worse clinical outcomes and mortality among COVID‐19 survivors [25, 26]. Pérez‐Catalán et al. reported that a high CCI negatively impacts physical functioning and general health [18], while Rodríguez‐Galán et al. observed that CCI negatively impacts HRQoL in individuals with post‐COVID‐19 pulmonary sequelae [27]. Santos et al. demonstrated that conditions such as hypertension, chronic kidney disease, chronic obstructive pulmonary disease, and cancer are associated with higher long‐term mortality in critically ill COVID‐19 patients [22]. Our findings support CCI as a predictor of both mortality and quality of life in COVID‐19 patients. Adequate management of comorbidities and prevention of underlying disease decompensation after discharge may mitigate the impact on quality of life and survival. Furthermore, our results indicated that the most common causes of death were pulmonary, cancer‐related, and cardiovascular complications, with approximately 50% of these deaths directly or indirectly related to COVID‐19. This suggests that COVID‐19 may have exacerbated existing comorbidities, in line with the data showing that, in 2021, COVID‐19 accounted for 24% of the total mortality rate in Brazil [28].

The severity of acute infection has consistently been shown to predict poorer quality of life and shorter long‐term survival in post‐COVID‐19 patients. In a Brazilian cohort, Rosa et al. found that patients requiring MV during hospitalization had lower quality of life scores and higher long‐term mortality rates [17]. Likewise, the study by Caamano et al. and the meta‐analysis by Gesser et al. identified acute illness severity as a predictor of poorer HRQoL [16, 24]. Our findings showed that the need for any level of oxygen therapy or ventilatory support was associated with poorer HRQoL, suggesting that even lower levels of oxygen therapy may significantly impact long‐term outcomes. This effect may be partly explained by the pulmonary function abnormalities and limited diffusion capacity observed in COVID‐19 survivors, as highlighted by Shah et al. [29] Conversely, while some studies suggest that lower oxygenation targets may be associated with better long‐term survival outcomes compared to higher targets, the evidence on long‐term mortality remains inconclusive [30, 31]. In our study, the association between the need for low oxygen therapy supplementation and mortality should be interpreted with caution, given the lack of association with higher flow supplementation strategies and ventilatory support, including MV. These results may have been influenced by survival bias, since our study sample consisted only of post‐hospitalization survivors.

Regarding BMI, our findings align with the obesity paradox often observed in other respiratory infections, where excess weight is associated with relative protection in critical illness [32]. However, this relationship with COVID‐19 remains to be established. While some studies suggest that obesity may increase the risk of adverse outcomes [33], this effect appears to be mediated by factors such as sex [34] or age [35].

### 4.1 Strengths and Limitations

Strengths of this study include its prospective cohort design, centralized 12‐month follow‐up, and focus on patient‐centered outcomes, ensuring high data quality and reducing potential reporting bias. This study also has several limitations. While our sample, representative of a middle‐income country with a high incidence of COVID‐19, enhances the relevance of the findings, the single‐country setting may limit their generalizability to contexts with different healthcare systems. We included several centers from almost all Brazilian regions, but most patients were from South and Southeast, introducing a bias toward more patients with a European ethnic background, but a high level of ethnic admixture is observed in the country.

Additionally, small subgroup sample sizes may have limited the statistical power of our analysis for certain secondary outcomes. To mitigate potential recall bias in the retrospective assessment of HRQoL before hospitalization (prior EQ‐5D‐3L score), the interviewer team received specialized training to assist participants in constructing a timeline of events, emphasizing aspects of quality of life before and after hospitalization. Finally, the sample consisted mostly of older adults with comorbidities, the majority of whom were vaccinated, which reflects the postvaccination context of the study and may influence the interpretation of the results.

## 5 Conclusion

In this multicentre cohort study, conducted during a period of Omicron predominance in Brazil, survivors of hospitalization for Omicron infection in Brazil experienced low HRQoL and high mortality at 1 year. Key factors associated with worse outcomes included older age, comorbidities, and the need for oxygen supplementation during hospitalization. These findings underscore the need for targeted interventions to mitigate long‐term impacts in vulnerable populations.

## Author Contributions

**Geraldine Trott:** conceptualization, methodology, project administration, resources, supervision, validation, visualization, writing – original draft. **Marciane Maria Rover:** conceptualization, investigation, methodology, supervision, validation, visualization, writing – review and editing. **Fernando Luis Scolari:** methodology, supervision, validation, writing – review and editing. **Mariana Motta Dias da Silva:** conceptualization, data curation, formal analysis, methodology, software, visualization, writing – review and editing. **Denise de Souza:** conceptualization, methodology, supervision, validation, visualization, writing – review and editing. **Rosa da Rosa Minho dos Santos:** conceptualization, investigation, methodology, supervision, validation. **Raine Fogliati De Carli:** investigation, supervision, validation. **Emelyn de Souza Roldão:** investigation, supervision, validation. **Gabriela Soares Rech:** investigation, supervision, validation. **Duane Mocellin:** investigation, validation. **Jennifer Menna Barreto de Souza:** investigation, validation. **Aline Paula Miozzo:** investigation, validation. **Carolina Rothmann Itaqui:** investigation. **Gabrielle Nunes da Silva:** investigation, validation, writing – review and editing. **Sergio Renato da Rosa Decker:** writing – review and editing. **Erica Neves Leite:** investigation, resources. **Carlos Delmar do Amaral Ferreira:** investigation, resources. **Lucas Gobetti da Luz:** investigation, resources. **Gabriel Beilfuss Rieth:** investigation, resources. **Lucas Tramujas:** investigation, resources. **Fernando Azevedo Medrado Jr:** investigation, resources. **Bruna Fornazieri Piotto:** investigation, resources. **Gizelle Fernanda Oliveira Silva:** investigation, resources. **Josafá Ferreira Chaves:** investigation, resources. **Saionara Cristina Francisco:** investigation, resources. **Precil Diego Miranda de Menezes Neves:** investigation, resources. **Victor Augusto Hamamoto Sato:** investigation, resources. **Viviane Cordeiro Veiga:** investigation, resources. **Kaique Lima Martins:** investigation, resources. **Cláudio Dornas de Oliveira:** investigation, resources. **Sabrina Gomes dos Santos:** investigation, resources. **Juliana Cardozo Fernandes:** investigation, resources. **Thiago Costa Lisboa:** investigation, resources. **Vivian Menezes Irineu:** investigation, resources. **Mauricio Antonio Pompilio:** investigation, resources. **Adriana de Oliveira França:** investigation, resources. **Aline Coletto Jaccottet:** investigation, resources. **Juliana Carvalho Schleder:** investigation, resources. **Vinicius Ortigosa Nogueira:** investigation, resources. **Vandack Nobre:** investigation, resources. **Daniel Souto Silveira:** investigation, resources. **Cézar Eumann Mesas:** investigation, resources. **Diego Miltersteiner:** investigation, resources. **Emanuelle Toledo Ortiz:** investigation, resources. **Fernando Gioppo Blauth:** investigation, resources. **Luciane Maria Facchi:** investigation, resources. **Milena Soriano Marcolino:** conceptualization, methodology, writing – review and editing. **Ana Carolina Peçanha Antonio:** conceptualization, methodology, writing – review and editing. **Paulo R Schvartzman:** conceptualization, methodology, writing – review and editing. **Bruna Brandão Barreto:** conceptualization, methodology, writing – review and editing. **Caroline Cabral Robinson:** conceptualization, methodology. **Maicon Falavigna:** conceptualization, methodology. **Carisi Anne Polanczyk:** conceptualization, methodology, writing – review and editing. **Luiz Antonio Nasi:** conceptualization, methodology. **Regis Goulart Rosa:** conceptualization, investigation, methodology, project administration, writing – review and editing; Post Covid Brazil 1 Group Authorship: (all authors contributed equally): investigation, resources. Included in Supporting Information e‐Table S5 are representatives from the 24 centers that included at least 1 patient.

## Ethics Statement

This study was conducted in accordance with the ethical standards of the Brazilian National Ethics Committee. Informed consent was obtained from all individual participants included in the study. Confidentiality of participant data was strictly maintained, and all personal identifiers were removed to ensure anonymity. Data collected from participants was used solely for the purposes of this study and will be stored securely in accordance with relevant data protection regulations.

## Consent

The authors have nothing to report.

## Conflicts of Interest

The authors declare that they have no competing interests.

## Supporting information


**e‐Figure 1.** Geographical distribution of the 24 participating centres: centres by state. **e‐Figure 2.** Radar chart of health‐related quality of life at each timepoint. **e‐Figure 3.** Comparison of participants' 12‐month EQ‐5D‐3L by ICU stay. **e‐Figure 4.** Multivariate generalised estimating equation (GEE) analysis of factors associated with quality of life at 12 months ‐ survivors only. **e‐Figure 5.** Kaplan‐Meier survival curve. **e‐Figure 6.** Causes of death. **e‐Table 1.** Comparison of baseline characteristics between participants included in and excluded from the primary outcome analysis. **e‐Table 2.** Reported problems across dimensions of the EQ‐5D‐3L among survivors at each timepoint. **e‐Table 3.** Mortality rate, causes of death, and relationship to COVID‐19 during follow‐up. **e‐Table 4.** Univariate survival analysis. **e‐Table 5.** Study organisation.

## Data Availability

The data that support the findings of this study are available from the corresponding author upon reasonable request.
