# Supplementary material for: One‐Year Quality of Life Among Survivors of Hospitalization for Omicron Infection in Brazil: A Multicentre Prospective Cohort Study
Source: J Med Virol. 2025 Nov 18;97(11):e70687. doi: 10.1002/jmv.70687 (PMC12624822; doi:10.1002/jmv.70687)
Supplement: Supplementary file 1 — e‐Figure 1. Geographical distribution of the 24 participating centres: centres by state. e‐Figure 2. Radar chart of health‐related quality of life at each timepoint. e‐Figure 3. Comparison of participants' 12‐month EQ‐5D‐3L by ICU stay. e‐Figure 4. Multivariate generalised estimating equation (GEE) analysis of factors associated with quality of life at 12 months ‐ survivors only. e‐Figure 5. Kaplan‐Meier survival curve. e‐Figure 6. Causes of death. e‐Table 1. Comparison of baseline characteristics between participants included in and excluded from the primary outcome analysis. e‐Table 2. Reported problems across dimensions of the EQ‐5D‐3L among survivors at each timepoint. e‐Table 3. Mortality rate, causes of death, and relationship to COVID‐19 during follow‐up. e‐Table 4. Univariate survival analysis. e‐Table 5. Study organisation. [file JMV-97-e70687-s001.docx]

**SUPPLEMENTARY MATERIAL**

**e-Table 1. Comparison of baseline characteristics between participants included in and excluded from the primary outcome analysis**

|  | **Included (N=619)** | **Excluded (N=30)** |
| --- | --- | --- |
| **Sex** |  |  |
| Female | 297/619 (47·98) | 17/30 (56·67) |
| **Age** |  |  |
| Under 60 years | 154/619 (24·88) | 9/30 (30·00) |
| Greater than or equal to 60 years | 465/619 (75·12) | 21/30 (70·00) |
| Median (IQR) | 71·00 (60·00-80·00) | 74·00 (54·50-84·50) |
| **Self-identified race^1^** |  |  |
| White | 489/619 (79·00) | 24/30 (80·00) |
| Non-White* | 130/619 (21·00) | 6/30 (20·00) |
| **Years of schooling, median (IQR)^2^** | 12·00 (7·00-16·00) | 12·00 (9·25-16·00) |
| **Smoking^3^** |  |  |
| Current smoking | 43/619 (6·95) | 1/30 (3·33) |
| Past smoking | 267/619 (43·13) | 13/30 (43·33) |
| No history of smoking | 309/619 (49·92) | 16/30 (53·33) |
| **Alcohol abuse^4^** | 90/619 (14·54) | 2/30 (6·67) |
| **Family income per month** |  |  |
| Up to R$2000 | 96/517 (18·57) | 5/22 (22·73) |
| R$2001 to R$4000 | 112/517 (21·66) | 2/22 (9·09) |
| R$4001 to R$10,000 | 121/517 (23·40) | 4/22 (18·18) |
| R$10,001 to R$20,000 | 96/517 (18·57) | 5/22 (22·73) |
| Above R$20,000 | 92/517 (17·79) | 6/22 (27·27) |
| **BMI** |  |  |
| BMI ≥ 25 | 165/619 (26·66) | 18/30 (60·00) |
| Median (IQR) | 25·97 (23·05-30·23) | 27·2 (23·96-30·4) |
| **CCI** |  |  |
| 0 | 179/619 (28·92) | 12/30 (40·00) |
| 1 | 183/619 (29·56) | 6/30 (20·00) |
| ≥ 2 | 257/619 (41·52) | 12/30 (40·00) |
| Median (IQR) | 1·00 (0·00-2·00) | 1·00 (0·00-2·00) |
| **Comorbidities** |  |  |
| Cardiovascular disease**^5^** | 356/619 (57·51) | 18/30 (60·00) |
| Systemic arterial hypertension | 280/619 (45·23) | 16/30 (53·33) |
| Diabetes | 210/619 (33·93) | 9/30 (30·00) |
| COPD, asthma, or pulmonary fibrosis | 137/619 (22·13) | 5/30 (16·67) |
| Solid tumour with and without metastasis | 80/619 (12·92) | 4/30 (13·33) |
| AIDS | 3/619 (0·48) | 1/30 (3·33) |
| History of mental illness**^6^** | 215/619 (34·73) | 10/30 (33·33) |
| **COVID-19 vaccination history** | 573/618 (92·72) | 28/29 (96·55) |
| **Worst severity scale score during hospital stay** |  |  |
| Without ventilatory support | 288/545 (52·84) | 13/20 (65·00) |
| Low-flow oxygen | 146/545 (26·79) | 5/20 (25·00) |
| Nasal cannula oxygen or NIV or MV | 111/545 (20·37) | 2/20 (10·00) |
| **Need for MV** | 25/618 (4·05) | 1/30 (3·33) |
| **Need for NIV** | 61/618 (9·87) | 2/30 (6·67) |
| **Need for high-flow nasal cannula** | 64/618 (10·36) | 0/30 (0·00) |
| **Need for low-flow oxygen** | 233/539 (43·23) | 6/20(30·00) |
| **Thromboembolic event during hospitalisation** | 25/618 (4·05) | 1/29 (3·45) |
| **Length of hospital stay, median (IQR)** | 8·00 (5·00-13·00) | 7·00 (5·00-9·00) |
| **ICU admission** | 115/619 (18·58) | 3/30 (10·00) |
| **Length of ICU stay** | 5·00 (2·00-10·00) | 2·00 (1·50-4·50) |

BMI: body mass index; CCI: Charlson comorbidity index; COPD: chronic obstructive pulmonary disease; ICU: intensive care unit; IQR: interquartile range (p25-p75); MV: mechanical ventilation; NIV: non-invasive ventilation.

Data are n/n total (%) unless otherwise specified.

**^1^** Non-White: those who self-identified as Black, Brown, Yellow, or Indigenous;

**^2^**Years of schooling are assessed without considering failing grades;

**^3^**Current smoking: having smoked at least 1 cigarette in the past 30 days;

**^4^**Alcohol abuse: 14 drinks per week for women and 21 drinks per week for men;

**^5^**Cardiovascular disease: heart failure, coronary artery disease with or without myocardial revascularisation, moderate/severe valve disease, resistant hypertension (3 or more antihypertensive drugs), permanent arrhythmias (atrial fibrillation/flutter), or cardiomyopathies;

**^6^**History of mental illness: previous diagnosis of anxiety or depression

**e-Table 2. Reported problems across dimensions of the EQ-5D-3L among survivors at each timepoint**

|  | **Prior (N=572)** | **3 months (N=596)** | **6 months (N=595)** | **9 months (N=587)** | **12 months (N=619)** |
| --- | --- | --- | --- | --- | --- |
| **Mobility,** **mean (SD)** | 612; 1·38 (0·55) | 549; 1·51 (0·60) | 536; 1·45 (0·58) | 517; 1·41 (0·58) | 536; 1·46 (0·62) |
| No problems | 403/612 (65·85) | 300/549 (54·64) | 318/536 (59·33) | 330/517 (63·83) | 329/536 (61·38) |
| Some problems | 187/612 (30·56) | 220/549 (40·07) | 194/536 (36·19) | 162/517 (31·33) | 170/536 (31·72) |
| Unable | 22/612 (3·59) | 29/549 (5·28) | 24/536 (4·48) | 25/517 (4·84) | 37/536 (6·90) |
| **Self-care, mean (SD)** | 612; 1·22 (0·54) | 549; 1·30 (0·60) | 536; 1·28 (0·59) | 517; 1·27 (0·57) | 536; 1·28 (0·60) |
| No problems | 516/612 (84·31) | 427/549 (77·78) | 425/536 (79·29) | 411/517 (79·50) | 427/536 (79·66) |
| Some problems | 60/612 (9·80) | 81/549 (14·75) | 71/536 (13·25) | 73/517 (14·12) | 66/536 (12·31) |
| Limited | 36/612 (5·88) | 41/549 (7·47) | 40/536 (7·46) | 33/517 (6·38) | 43/536 (8·02) |
| **Usual activities, mean (SD)** | 612; 1·29 (0·58) | 549; 1·51 (0·67) | 536; 1·45 (0·66) | 517; 1·38 (0·62) | 536; 1·43 (0·68) |
| No problems | 472/612 (77·12) | 326/549 (59·38) | 347/536 (64·74) | 358/517 (69·25) | 362/536 (67·54) |
| Some problems | 100/612 (16·34) | 167/549 (30·42) | 138/536 (25·75) | 120/517 (23·21) | 116/536 (21·64) |
| Unable | 40/612 (6·54) | 56/549 (10·20) | 51/536 (9·51) | 39/517 (7·54) | 58/536 (10·82) |
| **Pain/discomfort, mean (SD)** | 612; 1·52 (0·63) | 548; 1·62 (0·69) | 535; 1·64 (0·69) | 515; 1·55 (0·65) | 536; 1·55 (0·63) |
| No pain or discomfort | 342/612 (55·88) | 272/548 (49·64) | 257/535 (48·04) | 276/515 (53·59) | 280/536 (52·24) |
| Some pain or discomfort | 224/612 (36·60) | 212/548 (38·69) | 213/535 (39·81) | 195/515 (37·86) | 215/536 (40·11) |
| Extreme pain or discomfort | 46/612 (7·52) | 64/548 (11·68) | 65/535 (12·15) | 44/515 (8·54) | 41/536 (7·65) |
| **Anxiety/depression, mean (SD)** | 612; 1·49 (0·60) | 547; 1·61 (0·66) | 533; 1·58 (0·65) | 517; 1·56 (0·64) | 536; 1·55 (0·63) |
| Not anxious or depressed | 343/612 (56·05) | 265/547 (48·45) | 273/533 (51·22) | 273/517 (52·80) | 280/536 (52·24) |
| Moderately anxious or depressed | 236/612 (38·56) | 228/547 (41·68) | 212/533 (39·77) | 201/517 (38·88) | 217/536 (40·49) |
| Extremely anxious or depressed | 33/612 (5·39) | 54/547 (9·87) | 48/533 (9·01) | 43/517 (8·32) | 39/536 (7·28) |
| **EQ-5D-3L score, median (IQR)** | 0·74 (0·62-1·00) | 0·67 (0·42-0·80) | 0·68 (0·42-0·80) | 0·73 (0·42-1·00) | 0·69 (0·41-0·80) |

Data are n/n total (%) unless otherwise specified

IQR: interquartile range (p25-p75)

SD: standard deviation

**e-Table 3. Mortality rate, causes of death, and relationship to COVID-19 during follow-up**

| **Deaths** | **Total (N=619)** |
| --- | --- |
| Overall | 83/619 (13·41) |
| 3 months | 50/619 (8·08) |
| 6 months | 13/619 (2·10) |
| 9 months | 9/619 (1·45) |
| 12 months | 11/619 (1·78) |
| **Cardiovascular death** |  |
| No | 66/83 (79·52) |
| Yes | 14/83 (16·87) |
| Unable to assess | 3/83 (3·61) |
| **Cardiovascular causes** |  |
| Other | 7/14 (50·00) |
| Congestive heart failure | 4/14 (28·57) |
| Myocardial infarction | 3/14 (21·43) |
| **Other causes** |  |
| Pulmonary complications | 27/66 (40·91) |
| Cancer-related causes | 17/66 (25·76) |
| Neurological causes | 7/66 (10·61) |
| Non-COVID-19 infections | 6/66 (9·09) |
| Other | 5/66 (7·58) |
| Bleeding | 3/66 (4·55) |
| Gastrointestinal complications | 1/66 (1·52) |
| **Relationship to COVID-19*** |  |
| Not related | 42/83 (50·6) |
| Yes, directly or indirectly related | 34/83 (40·96) |
| Unable to assess | 4/83 (4·82) |
| Inconclusive | 3/83 (3·61) |

Data are n/n total (%).

Relationship to COVID-19*: adjudicators also evaluated whether a participant's death was associated with COVID-19 based on clinical criteria.

**e-Table 4. Univariate survival analysis**

| **Subgroups** | **HR** | **95% CI** | **p** |
| --- | --- | --- | --- |
| Female sex | 0·72 | (0·46;1·12) | 0·14 |
| Age ≥ 60 years | 4·38 | (1·91;10·07) | <0·001 |
| CCI = 1 | 1·83 | (0·91;3·70) | 0·09 |
| CCI ≥ 2 | 2·81 | (1·49;5·3) | 0·001 |
| BMI ≥ 25 kg/m² | 0·56 | (0·36;0·87) | 0·01 |
| Severity score 3^a^ | 2·35 | (1·37;4·04) | 0·001 |
| Severity score 4^b^ and 5^c^ | 1·72 | (0·92;3·23) | 0·08 |

95% CI: confidence interval; BMI: body mass index; CCI: Charlson comorbidity index; HR: hazard ratio.

^a^Severity score 3: admitted to hospital but requiring low-flow supplemental oxygen;

^b^Severity score 4: admitted to hospital requiring high-flow nasal cannula or non-invasive ventilation (NIV);

^c^Severity score 5: admitted to hospital requiring mechanical ventilation (MV) or extracorporeal membrane oxygenation (ECMO).

**e-Table 5. Study organisation**

***Participating centres***

| **Hospital Moinhos de Vento** | |
| --- | --- |
| Principal investigator | Carlos Delmar do Amaral - carlosferreira20362@gmail.com |
| Research team | Gabriel Bielfess Rieth  Vitoria Homem Machado  Lucas Gobetti da Luz  Janine Gonzaga  Andressa Daga  Laura Przybylski  Erica Leite  Danielle Amaral Pereira  Diogo Rocha |
| **Hospital Doutor Leo Orsi Bernardes de Itapetininga** | |
| Principal investigator | Vivian Menezes Irineu - virineu@gmail.com |
| Research team | Keillin Mirella de Almeida  Viviane Aparecida da Silva Correa  Mirian Aparecida Ferreira |
| **Hospital Ernesto Dornelles** | |
| Principal investigator | Juliana Cardozo Fernandes - jufernandes75@gmail.com |
| Research team | Tobias Cancian Milbradt  Bruna Lago |
| **Hospital Metropolitano Doutor Célio de Castro** | |
| Principal investigator | Saionara Cristina Francisco - saionaracf@gmail.com |
| Research team | Gizelle Fernanda Oliveira Silva  Josafá Ferreira Chaves |
| **Hospital Alemão Oswaldo Cruz** | |
| Principal investigator | Precil Diego Miranda de Menezes Neves - pmenezes@haoc.com.br |
| Research team | Victor Augusto Hamamoto Sato  Marina Andrade Faria  Antonio Marcos Assuncim  Rose Cristina Moreira Silva  Roberta Barbuio Macota de Souza  Letícia Issa Lledo  Mariana de Antonio Corradi |
| **Hospital do Coração** | |
| Principal investigator | Lucas Tramujas |
| Research team | Fernando Azevedo Medrado Junior  Bruna Fornazieri Piotto  Letícia Barbante |
| **Hospitais da Universidade Estadual de Ponta Grossa** | |
| Principal investigator | Juliana Carvalho Schleder - juliana.schleder@uepg.br |
| Research team | Pollyanna Kassia de Oliveira Borges  Ricardo Zanetti Gomes |
| **Hospital e Maternidade SEPACO** | |
| Principal investigator | Flávio Freitas - fgrfreitas@sepaco.org.br |
| Research team | Daniela Boschetti  Nathaly Fonseca Nunes |
| **Instituto Orizonti** | |
| Principal investigator | Estevão Lanna Figueiredo - estevao@cardiol.br |
| Research team | Fernando Carvalho Neuenschwander |
| **Hospital Beneficência de Ribeirão Preto** | |
| Principal investigator | Fernando Gioppo Blauth - blauthfernando@gmail.com |
| Research team |  |
| **Hospital Universitário de Canoas** | |
| Principal investigator | Diego Miltersteiner - md.diegorm@gmail.com |
| Research team | Emanuelle Toledo Ortiz |
| **Hospital do Coração de Mato Grosso do Sul** | |
| Principal investigator | Mauricio Antonio Pompilio - mapompilio@yahoo.com.br |
| Research team | Adriana de Oliveira França |
| **Hospital Vila Nova** | |
| Principal investigator | Luciane Maria Facchi - lufacchi@uol.com.br |
| Research team |  |
| **Hospital de Clínicas da Universidade Federal de Minas Gerais** | |
| Principal investigator | Vandack Alencar Nobre Jr - vandack@gmail.com |
| Research team |  |
| **Hospital Beneficência Portuguesa de São Paulo** | |
| Principal investigator | Viviane Cordeiro Veiga - viviane.veiga@bp.org.br |
| Research team | Kaique Lima Gomes  Juliana Chaves Coelho  Felipe Augusto da Silva  Elisangela da Silva Rodrigues Marçal |
| **Centro de Medicina Tropical de Rondônia** | |
| Principal investigator | Vinicius Ortigosa Nogueira - vinicius.nogueira@unir.br |
| Research team | Manuela Marinho de Andrade  Solana Monteiro Batista |
| **Hospital Universitário de Londrina** | |
| Principal investigator | Cézar Eumann Mesas - cemesas@uel.br |
| Research team | Daniela dos Anjos |
| **Instituto de Cardiologia - Fundação Universitária de Cardiologia** | |
| Principal investigator | Aline Coletto Jaccottet - aline.coletto@hotmail.com |
| Research team | Maico Furlanetto  Guilherme Antônio Viganó |
| **Hospital de Clínicas de Porto Alegre** | |
| Principal investigator | Thiago Costa Lisboa - tlisboa@hcpa.edu.br |
| Research team | Ana Carolina Mardini  Andressa Daga  Patrícia Fernandes |
| **Centro de Pesquisa Clínica e Populacional Hospitaliza - Nelson Cornetet** | |
| Principal investigator | Daniel Souto Silveira - danielsscardio@hotmail.com |
| Research team | Letícia Klein  Cláudia Teixeira |
| **Hospital São Vicente de Paulo** | |
| Principal investigator | Alexandre Pereira Tognon - aptognon@hsvp.com.br |
| Research team | Aloma Santin Menegasso |
| **Hospital Emydio Germano - Santa Casa de Belo Horizonte** | |
| Principal investigator | Cláudio Dornas de Oliveira - claudiodornas@santacasabh.org.br |
| Research team | Sabrina Gomes dos Santos  Isabela Olegário Bernardes Louredo  Daiane Joice Ferraz Santos  Deiverson Richard Soares Menezes  Ariadne Poliana de Oliveira Bastos  Carolina Thais Pires Costa |
| **Hospital de Clínicas de Passo Fundo** | |
| Principal investigator | Janaina Pilau - jpilau@hotmail.com |
| Research team | Priscila Tonial Foscarini |

|  | Name |
| --- | --- |
| Steering committee | Milena Soriano Marcolino |
|  | Bruna Brandão Barreto |
|  | Paulo R Schvartzman |
|  | Ana Carolina Peçanha Antonio |
|  | Caroline Cabral Robinson |
|  | Maicon Falavigna |
|  | Carisi Anne Polanczyk |
|  | Regis Goulart Rosa |
|  | Marciane Maria Rover |
|  | Geraldine Trott |
| Investigators | Geraldine Trott |
|  | Marciane Maria Rover |
|  | Fernando Luis Scolari |
|  | Denise de Souza |
|  | Ana Paula Aquistapase Dagnino |
|  | Rosa da Rosa Minho dos Santos |
|  | Mariana Motta Dias da Silva |
|  | Raíne Fogliati de Carli |
|  | Gabriela Soares Rech |
|  | Emelyn de Souza Roldao |
|  | Duane Mocellin |
|  | Aline Paula Miozzo |
|  | Jennifer Menna Barreto de Souza  Gabrielle Nunes da Silva |
|  | Carolina Rothmann Itaqui |
|  | Gabriel Pozza Muller Estivalete |
|  | Juliana de Mesquita Neto |
|  | Hellen Jordan Martins Freitas |
|  | Adriana Avila |
|  | Catherine Vitoria Pereira dos Santos |
|  | Christian Morais Soares |
|  | João Vítor Gozzi |
|  | Ingrid Flor dos Santos |
|  | Carla Moura D´Ávila |
|  | Camilli Galvão Mooler |
|  | Alanys Santos da Silveira |
|  | Love Lamantha Morain |

**e-Figure 1. Geographical distribution of the 24 participating centres: centres by state**


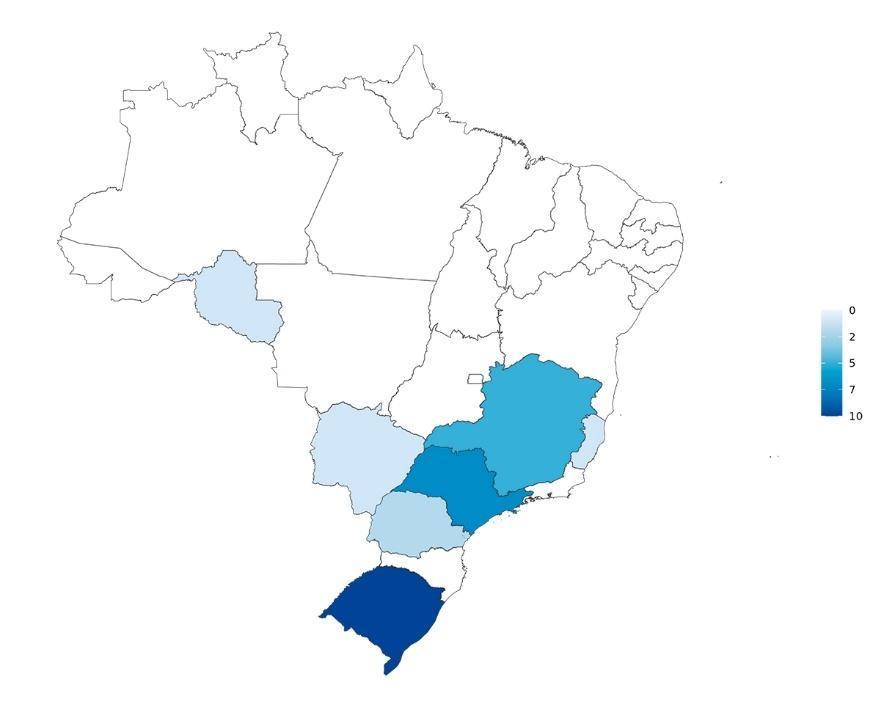


**e-Figure 2. Radar chart of health-related quality of life at each timepoint**


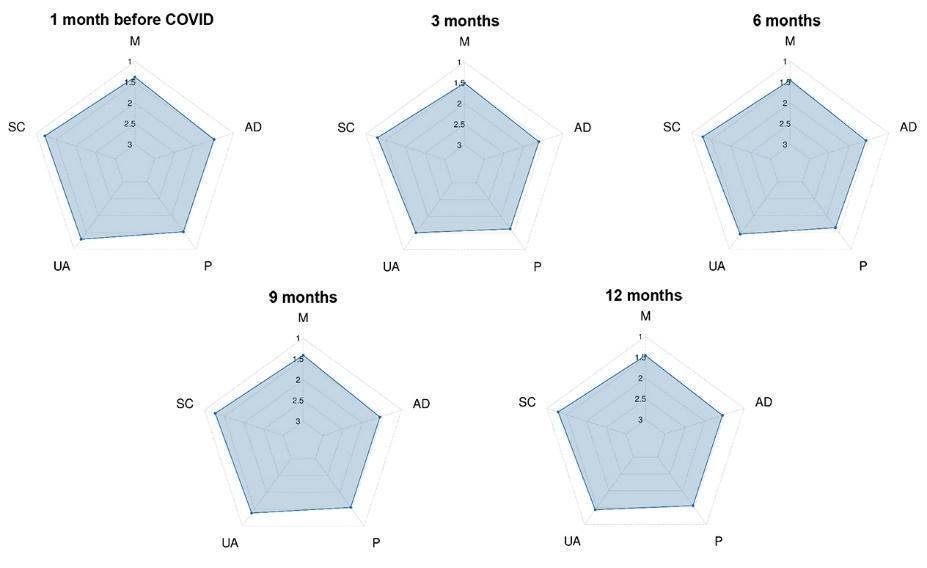


AD: anxiety/depression

M: mobility

P: pain

SC: self-care

UA: usual activities

**e-Figure 3. Comparison of participants' 12-month EQ-5D-3L by ICU stay**


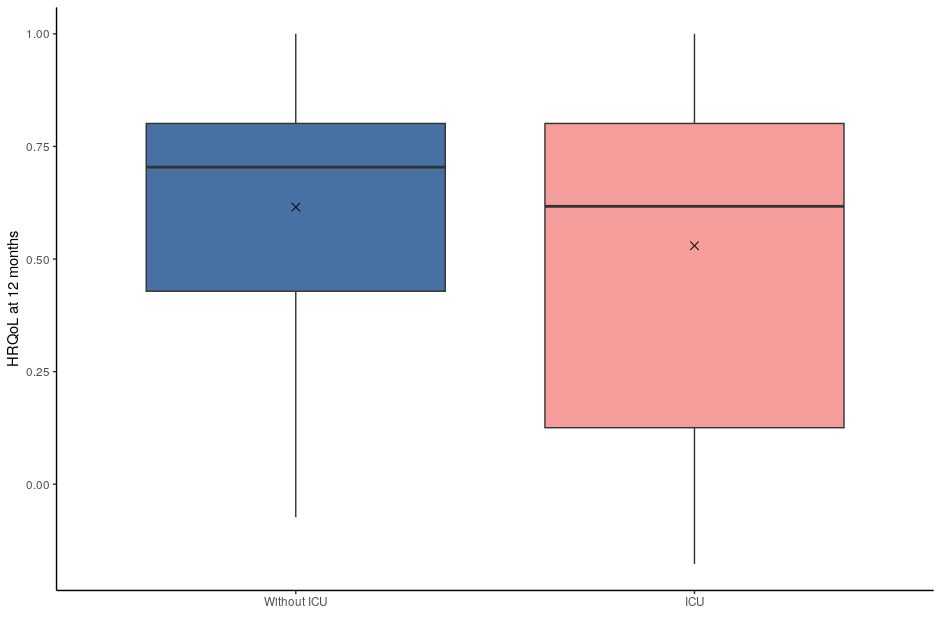


EQ-5D-3L, EuroQol five-dimension three-level questionnaire.

The x marker represents mean, the box plot inner horizontal lines represent median. Boxes represent IQR (25th and 75th percentiles), vertical whiskers represent 1·5 IQR beyond the 25th and 75th percentiles; extreme values were not represented in the figure. In the Brazilian population, EQ-5D-3L scores range from -0·17 (worst) to 1 (best), with a minimal clinically important difference of 0·03.^11^ The mean value in the Brazilian population is 0·82.^12^ Patients who died during follow-up were assigned a score of zero on all subsequent follow-ups.

**e-Figure 4. Multivariate generalised estimating equation (GEE) analysis of factors associated with quality of life at 12 months - survivors only**


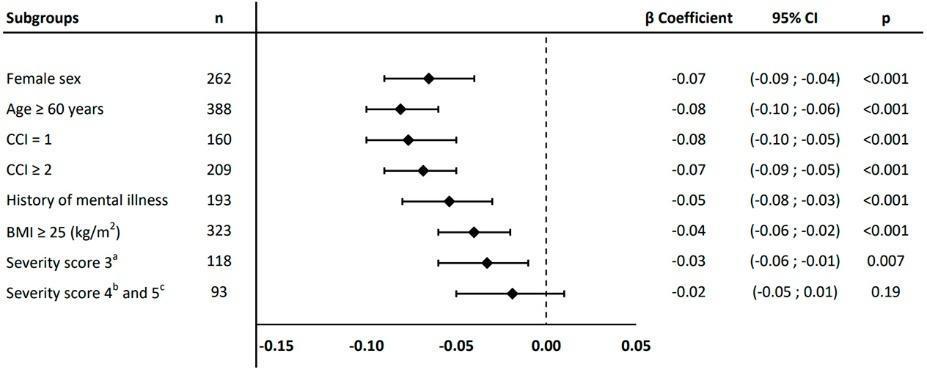


95% CI: confidence interval; BMI: body mass index; CCI: Charlson comorbidity index

History of mental illness: previous diagnosis of anxiety or depression

^a^Severity score 3 - admitted to hospital but requiring low-flow supplemental oxygen;

^b^Severity score 4 – admitted to hospital requiring high-flow nasal cannula or non-invasive ventilation (NIV);

^c^Severity score 5 – admitted to hospital requiring mechanical ventilation (MV) or extracorporeal membrane oxygenation (ECMO).

β: The beta coefficient represents the expected change in the dependent variable for a one-unit change in the independent variable, while maintaining all other variables constant.

**e-Figure 5 - Kaplan-Meier survival curve**

**
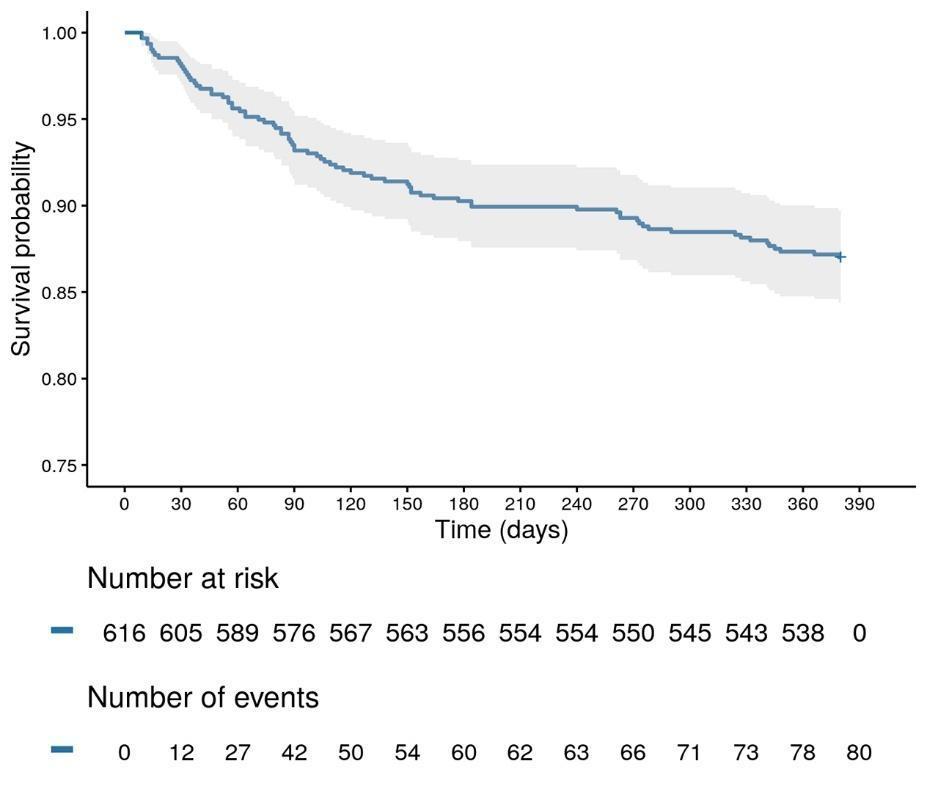
**

**e-Figure 6. Causes of death**

**
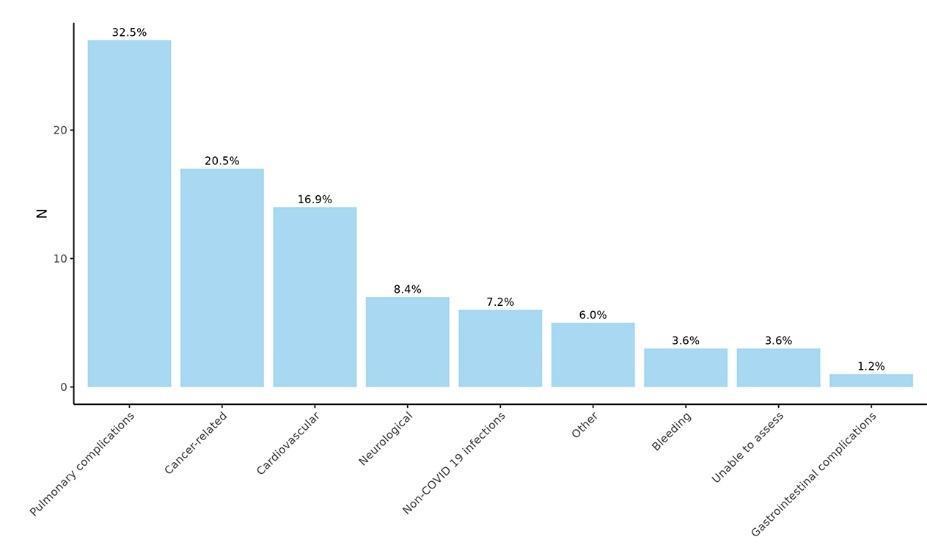
**
